# Preparation, Characterization and Application of a Molecularly Imprinted Polymer for Selective Recognition of Sulpiride

**DOI:** 10.3390/ma10050475

**Published:** 2017-04-28

**Authors:** Wei Zhang, Xuhui She, Liping Wang, Huajun Fan, Qing Zhou, Xiaowen Huang, James Z. Tang

**Affiliations:** 1 School of Basic Courses, Guangdong Pharmaceutical University, Guangzhou 510006, China; gzzhangw@163.com; 2 School of Pharmacy, Guangdong Pharmaceutical University, Guangzhou 510006, China; lab-shexuhui@kingmed.com.cn (X.S.); wangjiang0916@126.com (L.W.); kytxor@163.com (Q.Z.); xiaowenh25@126.com (X.H.); 3 Guangzhou KingMed Center for Clinical Laboratory Co., Ltd., Guangzhou 510005, China; 4 China National Analytical Center Guangzhou, Guangzhou 510070, China; 5 Faculty of Science and Engineering, University of Wolverhampton, Wolverhampton WV1 1LY, UK; J.Z.Tang@wlv.ac.uk

**Keywords:** molecularly imprinted polymer, sulpiride, itaconic acid, serum analysis, drug release

## Abstract

A novel molecular imprinting polymer (MIP) was prepared by bulk polymerization using sulpiride as the template molecule, itaconic acid (ITA) as the functional monomer and ethylene glycol dimethacrylate (EGDMA) as the crosslinker. The formation of the MIP was determined as the molar ratio of sulpiride-ITA-EGDMA of 1:4:15 by single-factor experiments. The MIP showed good adsorption property with imprinting factor *α* of 5.36 and maximum adsorption capacity of 61.13 μmol/g, and was characterized by scanning electron microscopy (SEM), Fourier-transform infrared spectroscopy (FT-IR) and surface area analysis. With the structural analogs (amisulpride, tiapride, lidocaine and cisapride) and small molecules containing a mono-functional group (p-toluenesulfonamide, formamide and 1-methylpyrrolidine) as substrates, static adsorption, kinetic adsorption, and rebinding experiments were also performed to investigate the selective adsorption ability, kinetic characteristic, and recognition mechanism of the MIP. A serial study suggested that the highly selective recognition ability of the MIP mainly depended on binding sites provided by N-functional groups of amide and amine. Moreover, the MIP as solid-phase extractant was successfully applied to extraction of sulpiride from the mixed solution (consisted of p-toluenesulfonamide, sulfamethoxazole, sulfanilamide, p-nitroaniline, acetanilide and trimethoprim) and serum sample, and extraction recoveries ranged from 81.57% to 86.63%. The tentative tests of drug release in stimulated intestinal fluid (pH 6.8) demonstrated that the tablet with the MIP–sulpiride could obviously inhibit sulpiride release rate. Thus, ITA-based MIP is an efficient and promising alternative to solid-phase adsorbent for extraction of sulpiride and removal of interferences in biosample analysis, and could be used as a potential carrier for controlled drug release.

## 1. Introduction

Sulpiride is a type of benzamide antipsychotic medication for schizophrenia. Chemically, it is also a substituted benzamide derivative related to metoclopramide and trimethobenzamide. As an antipsychotic drug of the benzamide class, sulpiride is mainly used in the treatment of psychosis associated with schizophrenia and major depressive disorder, and sometimes used in low dosage to treat anxiety and mild depression [1,2,3]. Recently, schizophrenia has a significant rise with China’s economic booming, thus sulpiride is widely used in clinical applications and administrated in large dose for typical acute treatment [1,4,5,6,7]. In addition, sulpiride as a relative old antipsychotic drug is more cost-effective than newer drugs in developing countries, and also has had other uses including treatment of peptic ulcer, vomiting and vertigo. To avoid overdose and side effects of sulpiride in clinical applications, monitoring sulpiride, especially in China, has become increasingly demanded for mental diseases.

Molecularly imprinted polymers (MIPs) have been widely developed for separations, sensors, catalysis, biological mimics and other fields due to the specific molecular recognition of small and/or large molecules and stable physichemical properties [8,9,10,11,12,13,14,15]. Considering their specific adsorption, MIPs used as carrier materials have great potential in clinical and pharmaceutical applications such as biosample analysis and drug delivery [16,17,18,19]. For the preparation of MIPs, the selection of functional monomers is critical to the molecular recognition ability. An appropriate functional monomer can not only improve the affinity of the template molecule but also reduce the nonspecific adsorption of the MIP to enhance its specific recognition to the template molecule [16,20,21]. In previous studies, the MIP using methacrylic acid (MAA) as functional monomer exhibited good selective adsorption to sulpiride, but also maintained higher capacity for nonspecific adsorption [22,23]. In consideration of the chemical structure, itaconic acid (ITA) as four-carbon dicarboxylic acid can provide two carboxyl groups to a molecule of sulpiride when coming together and interacting with each other. Compared with freer MMA, ITA is more likely to match sulpiride in space to reduce non-specific adsorption. In our pre-experiment, we also found that using ITA as the functional monomer can decrease significantly the adsorption amount of the non-imprinted polymers (NIP) by at least half compared to that of the MMA-based NIP. Thus, our new approach is to prepare a MIP using ITA as the functional monomer with ethylene glycol dimethacrylate (EGDMA) as crosslinking agent, and its formulation conditions were optimized by single-factor experiment for enhancement of the specific adsorption. Characterization of the made MIP was performed using Fourier Transform Infrared (FT-IR), scanning electron microscope (SEM) and Brunauer–Emmett–Teller (BET) surface area analysis for the connection with monomer/template interaction, structural morphologies and surface parameters, respectively. To evaluate the adsorption capacity and selectivity of the MIP, adsorption properties were systematically investigated by static equilibrium adsorption and isothermal adsorption experiments in progression from sulpiride, its analogs and a mixture of sulpiride and its analogs. Moreover, the recognition mechanism was tentatively studied by adsorption tests of p-toluenesulfonamide, formamide and 1-methylpyrrolidine with individual functional groups consisting of sulpiride. In further consideration of medicine uses, we attempted to apply the MIP obtained to analysis of sulpiride in serum complex matrix, and the release features of the MIP loading sulpiride and its tablet were also investigated in controlled drug release.

## 2. Materials and Methods

### 2.1. Materials and Reagents

Sulpiride was purchased from Advanced Technology & Industrial Co., Ltd. (Hong Kong, China). Itaconic acid (ITA) was obtained from Shanghai Jing Chun Pty Ltd (Shanghai, China). Ethylene glycol dimethacrylate (EGDMA) was purchased from Eternal Chemical Co., Ltd. (Taiwan). Azoisobutyronitrile (AIBN) was bought from Tianjin Bai Shi Chemical Co., Ltd (Tianjin, China). Acetonitrile (chromatography grade) was purchased from Merck (Darmstadt, Germany). All the other reagents were analytical grade.

Sulpiride stock and standard solutions: Sulpiride (34.1 mg, 0.10 mmol) was weighed, dissolved and diluted up to the volume of 100 mL with methanol in a volumetric flask. Then, 1.00 mmol/L of the stock solution was sealed properly and stored in a fridge. The working standard solution was gradually diluted from the stock solution when used.

### 2.2. Preparation of the MIP

The template molecule sulpiride (102.4 mg, 0.3 mmol) and the functional monomer ITA (156.1 mg, 1.2 mmol) were added to 6 mL of methanol, and sonicated for 30 min. The crosslinker (EGDMA) (892.0 mg, 4.5 mmol) and the initiator (AIBN) (60.0 mg, 0.37 mmol) were was added to the mixture above, and sonicated for 5 min until well mixed. Under nitrogen protection, the polymerization was carried out at 60 °C for 24 h in a water bath (HH-6, Jintan, China). White bulk polymer was collected, ground and sieved through a 200 mesh sieve (particle size of 74 μm or below).

MIP powder was refluxed in a Soxhlet apparatus with methanol-acetic acid (9:1 v/v) for removal of the sulpiride. The reflux eluents were detected in intervals at 234 nm by UV-Vis spectrophotometer until no sulpiride was found. The MIP was further washed with methanol to remove residual acetic acid, and dried at 60 °C under vacuum for 12 h. Reference non-imprinted polymers (NIPs) were prepared under identical conditions without the presence of sulpiride; the obtained polymers were ground in a mortar, and then passed through a 200 mesh sieve. MIP and NIP powders prepared according to the above procedure were used for the following experiments.

### 2.3. Material Characterization [21]

FT-IR spectra of the MIP and the NIP were obtained by FT-IR analysis. KBr disks of the MIP and the NIP were respectively prepared, and their spectra were recorded at 4000 cm^−1^–400 cm^−1^ on a WQF-410FT FT-IR spectrometer (Beijing, China). Electron micrographs were taken for evaluation of the morphology with a Zeiss EVO 18 scanning electron microscope (SEM) (Carl-Zeiss-Strasse, Oberkochen, Germany). Sample powder was attached to the sample holder, dried and sputtered with gold in a SBC-12 Ion Sputtering Coater (Beijing, China). Morphologies of the MIP and the NIP were observed under SEM scanning at the voltage of 20 kV. The specific surface area, pore volume and pore size distribution of polymers were measured by a surface area analyzer (NOVA4200e, Quantachrome, Boynton Beach, FL, USA). Then, 0.1 g of sample was outgassed under vacuum for 6 h at 100 °C. The surface area and pore size were determined at 77 K by N_2_ adsorption and desorption isotherms using multipoint Brumauer-Emmett-Teller (BET) method.

### 2.4. Static Adsorption of the MIP

Static adsorption of the MIP and the NIP was carried out according to the following procedure. Briefly, the MIP or NIP (20.0 mg) was added into a conical flask with 2.0 mL of 1.0 mmol/L sulpiride (or other substrate) methanol solution. The flask sealed was shaken at 30 ℃ for 3 h in an oscillator. The concentration of the final sulpiride solution was determined in triplicate at a 234 nm on UV-Vis spectrophotometer [23,24]. The binding capacity of the MIP or NIP was calculated by Equation (1):

*Q* = (*C*_0_ − *C*_t_) *V*/*m* (1)

where *Q* is the binding capacity (μmol/g), *C_0_* is the initial concentration of sulpiride (μmol/L), *C*_t_ is the concentration of sulpiride at time t (μmol/L), *V* is the volume of the initial sulpirde solution (L) and *m* is the mass of the MIP or the NIP (g).

The imprinting factor was calculated by Equation (2):

*α* = *Q*_MIP_/*Q*_NIP_ (2)

where *α* is imprinting factor, *Q*_MIP_ is the adsorption capacity of the MIP (μmol/g), and *Q*_NIP_ is the adsorption capacity of the NIP (μmol/g).

The specificity adsorption ratio was calculated by Equation (3):

Specific adsorption ratio (%) = [(*Q*_MIP_ − *Q*_NIP_)/*Q*_MIP_] × 100% (3)

The selectivity of the MIP was evaluated by the specific factor *β*, which was defined as the ratio of the difference of the adsorption capacity between the MIP and NIP by a substrate against sulpiride. The specific factor *β* was calculated using Equation (4):

*β* = *Q*_substrate_/*Q*_sulpiride_ (4)

where *Q_s_*_ubstrate_ = (*Q*_MIP_ − *Q*_NIP_)_substrate_ and *Q_s_*_ulpiride_ = (*Q*_MIP_ – *Q*_NIP_)_sulpiride_. Both were characterized by exploiting the following procedure.

### 2.5. Solid-Phase Extraction of the MIP

Briefly, the MIP or NIP (20.0 mg) was added into a conical flask with 2.0 mL of a mixed substrate methanol solution (containing 0.20 mmol/L of sulfamethoxazole, sulfanilamide, p-toluenesulfonamide, p-nitroaniline, acetanilide, trimethoprim and sulpiride,). The flask sealed was shaken at 30 °C for 3 h. The resulting solution was filtered and diluted with methanol, then the concentration was determined at 250 nm at room temperature by an Agilent 1200 HPLC with UV detector (Agilent, Santa Clara, CA, USA) using a Gemini C_18_ column (4.6 mm × 250 mm, 5 µm, Phenonmenex). The mobile phase was comprised of methanol (A) and 0.1% ammonia solution (pH = 10.3) (B) according to the following gradient mode: 0–4 min, 18% A (v/v); 4–10 min, 18%–40% A (v/v); 10–25 min, 40%–48% A (v/v); 25–26 min, 48%–18% A (v/v). The flow rate was 1.0 mL/min. The injection volume was 20 µL.

### 2.6. The Preparation and Analysis of Serum Samples

Rat serum (1.0 mL) containing sulpiride was added with 1.0 mL of acetonitrile and mixed well. The mixture was centrifuged for 15 min. The filtrate was collected and evaporated. The obtained residue was dissolved in 1.0 mL of methanol, where 10 mg of the MIP powder was added to extract sulpiride. The mixture was shaken at 30 °C for 3 h in an oscillator. The loaded MIP was collected by filtration and then eluted with methanol-acetic acid (9:1). The collected wash-out was evaporated to dryness. After the residue was redissolved with methanol, sulpiride extracted from the MIP was determined at 234 nm under room temperature by HPLC using a Gemini C_18_ column (4.6 mm × 250 mm, 5 µm, Phenonmenex) as the stationary phase. The mobile phase was made of methanol and 0.1% ammonia solution (pH = 10.3) (45:55, v/v). The flow rate was 1.0 mL/min, and injection volume was 20 µL.

### 2.7. Drug Loading and Preparation of MIP-Sulpiride Tablets

Drug loading: MIP–sulpiride was prepared by loading with sulpiride in the MIP. Briefly, 0.25 g MIP powder was added in a conical flask with 15 mL of 2.5 mmol/L sulpiride methanol solution, then placed and shook in an oscillator at 30 °C for 12 h. The mixture was filtrated by 0.45 μm membrane, and the MIP was dried in vacuo overnight. The filtrate was analyzed by UV spectrophotometry to determine sulpiride in the MIP, and its loading capacity was determined as 30.69 μg/mg.

Preparation of tablets: MIP tablets were made by dry granulation tabletting using MIP–sulpiride and pharmaceutical excipients such as microcrystalline cellulose (MCC), hydroxy propyl methyl cellulose (HPMC), ethyl cellulose (EC) and povidone K30 (PVP-K30) according to the following procedure. Twenty milligrams of MIP–sulpiride powder (containing 613.8 μg of sulpiride) was well mixed with 200 mg of MCC, 40 mg of HPMC, 25 mg of EC and 15 mg of PVP-K30, and then made into tablets on a DP single-punch tablet press (Shanghai, China). For comparison, sulpiride control tablets were prepared according to the same procedure but without the MIP.

### 2.8. Drug Release of the MIP

Triplicates of MIP–sulpiride or a tablet prepared above were respectively added in stoppered conical flasks (250 mL) with 100 mL of 0.1 mol/L HCl buffer (pH 1.0, simulating the gastric fluid), KH_2_PO_4_-Na_2_HPO_4_ buffer (pH 6.8, simulating the intestinal fluid) and KH_2_PO_4_-Na_2_HPO_4_ buffer (pH 7.4, simulating the blood plasma. Controlled release of sulpiride was carried out at 37 ± 0.5 °C and 100 rpm in a ZRS-8G dissolution tester (Tianjin, China). Certain volumes of solutions were withdrawn at appropriate time intervals, and thus successively determined amounts of drug released by HPLC. The cumulative release rate (%) can be calculated by the following equation:

Cumulative release rate (%) = (*M*_t_/*M*_∞_) × 100 (5)

where *M_t_* is the accumulative amount of sulpiride at a certain moment, and *M_∞_* is the accumulative amount of sulpiride at over an infinite time.

## 3. Results and Discussion

### 3.1. Polymerization Conditions of MIPs

**Figure 1:**
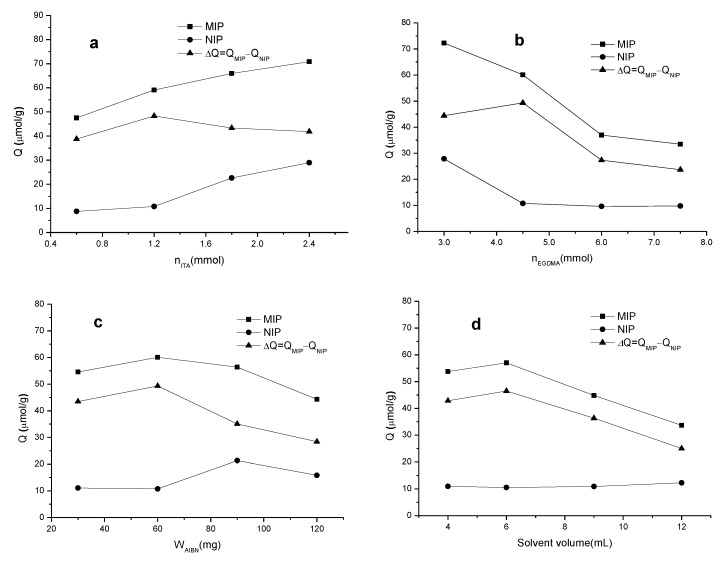
Optimization of the molecularly imprinted polymer (MIP) formulations against the adsorption capacity of the MIP and the non-imprinted polymer (NIP): (**a**) itaconic acid (ITA); (**b**) ethylene glycol dimethacrylate (EGDMA); (**c**) azoisobutyronitrile (AIBN); and (**d**) methanol.

The MIP was formulated with template-sulpiride, monomer-ITA, crosslinker-EGDMA, initiator-AIBN, and solvent-methanol. Although the molar ratio of template/monomer/crosslinker was empirically set at 1:4:15 [13,21,22], optimization of MIP formulations was carried out and assessed against the adsorption capacity of the MIP formed using single-factor experiment. The results, as shown in Figure 1, demonstrated the effects of amounts of ITA, EGDMA, AIBN and methanol on the adsorption capacity of the MIP and the NIP. The adsorption amounts of sulpiride in the MIP and the NIP increased with ITA, but the excess of ITA did not translate into the specific adsorption, *Q*_sulpiride_ [*Q*_sulpiride_ = (*Q*_MIP_ – *Q*_NIP_)_sulpiride_]. Similarly, the adsorption amounts of sulpiride decreased with increase of EGDMA, whereas the hardness of the MIP increased. More AIBN, otherwise, produces finer particles, resulting in loss of *Q*_sulpiride_. Methanol (6 mL) as solvent is the best choice in terms of the specific adsorption. Thus, optimization polymerization conditions suggested that 0.3 mmol of sulpiride, 1.2 mmol of ITA, 4.5 mmol of EGDMA, 60 mg of AIBN and 6 mL of methanol for a MIP formulation, corresponding to the molar ratio of 1:4:15 for template-sulpiride/monomer-ITA/crosslinker-EGDMA, achieving a best value of specific adsorption. Recent publications frequently used the molar ratio of 1:4 although monomer could be ranging from 1 to 10 times the template in mole [25,26,27,28,29,30,31,32,33,34,35].

### 3.2 Characterization of the MIP

**Figure 2:**
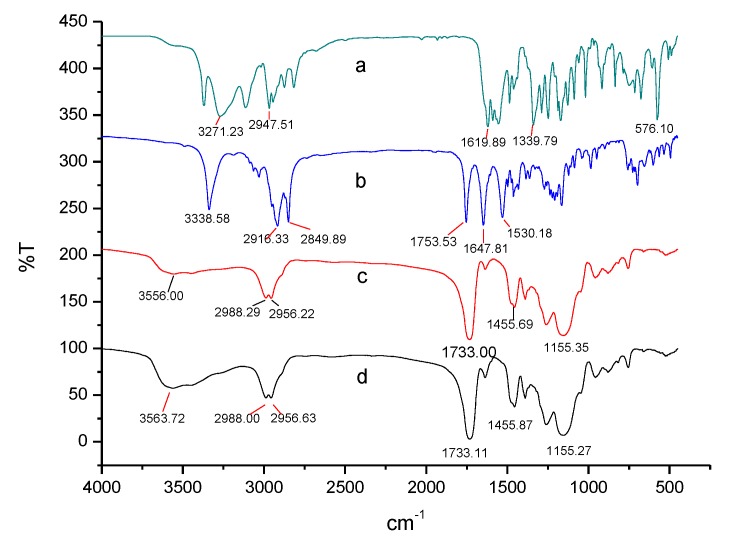
Fourier Transform Infrared (FT-IR) spectra of the MIP and the NIP: (**a**) sulpiride; (**b**) MIP with sulpiride; (**c**) NIP; and (**d**) MIP.

Sulpiride had characteristic absorption bands at 3380 cm^−1^ for N-H of the sulfonamide group, 3080 cm^−1^ for C–H of the aromatic ring, 1645 cm^−1^ for C=O of amide I, and 1550 cm^−1^ for N–H of amide II [21,36]. The NIP absent of sulpiride has no N-H related amide I and II and aromatic ring. Figure 2 shows FT-IR spectra of the MIPs before and after elution against the NIP. FT-IR spectra illustrated that the MIP after elution was almost identical to the NIP, which indicated the template was removed from the polymer matrix. The MIP before elution had comparable sulpiride absorption bands at 3338 cm^−1^, 1753 cm^−1^, 1647 cm^−1^ and 1530 cm^−1^, but the above characteristics obviously weakened or disappeared after elution. Asymmetric stretching of C–O–C, asymmetric deformation peak of C–H and symmetric stretching of C=O were, respectively, at 1155 cm^−1^, 1455 cm^−1^ and 1733 cm^−1^, exhibiting structural characteristics of EGDMA. The presence of the above characteristic peaks demonstrated that MIP was successfully synthesized.

**Figure 3:**
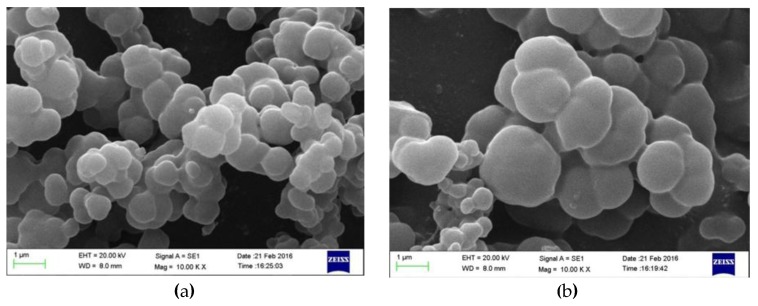
Scanning electron micrographs of: the MIP (**a**); and the NIP (**b**).

To evaluate surface characteristics, the morphology and surface parameters of the MIP and NIP were further investigated by scanning electron microscope (SEM) and Brumauer-Emmett-Teller (BET) method. From the SEM micrographs shown in Figure 3, both polymers exhibited a rough and microporous structure. Moreover, specific surface area, pore volume and pore size of the MIP and NIP were also measured through N_2_ adsorption–desorption analysis; the results were 17.035 m^2^/g, 0.035 cm^3^/g, and 6.808 nm for the MIP, and 12.543 m^2^/g, 0.025 cm^3^/g, 5.805 nm for the NIP, respectively. Thus, the MIP showed obviously larger specific surface area and more multiporous structure compared with the NIP, implying that it facilitated adsorption of sulpiride.

### 3.3. Adsorption Characteristics of the MIP

#### 3.3.1. Adsorption Performance of the MIP

**Figure 4:**
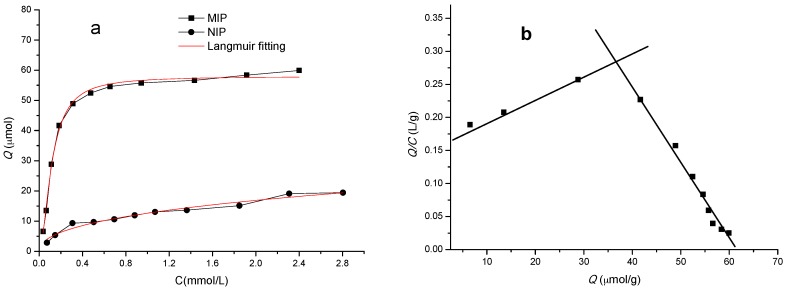
The adsorption isotherms of the MIP and the NIP (**a**); and Scatchard profile of the MIP (**b**).

The adsorption isotherm of the MIP and/or NIP can be obtained by adding incremental amounts of template to a given amount of the MIP and/or NIP. In addition, Scatchard analysis model was used for the evaluation of the adsorption of the MIP [37]. In Figure 4a, the adsorption amounts of polymers increased with the increase of sulpiride concentration. Relatively, the adsorption capacity of the MIP is much higher than that of the NIP, indicating that the MIP offered a stronger affinity to the template than the NIP. The equilibrium data were well represented by the Langmuir adsorption model I [38,39], and the MIP showed higher fitting degree with correlation coefficients (R^2^) of 0.9948.

The Scatchard analysis in Figure 4b follows the equation:

*Q/C* = (*Q*_max_-*Q*)/*K*_d_ (6)

where *Q* is the equilibrium adsorption capacity of sulpiride (μmol/g), *K*_d_ is the equilibrium dissociation constant (μmol/L) at the binding sites, *Q*_max_ is the maximum apparent adsorption at the binding sites (μmol/g) and *C* is the concentration of sulpiride in the solution (mmol/L). Figure 4b indicates that there are two segments in the curve corresponding to low concentration range and high concentration range of sulpiride. The equations of the two segments are:

*Q/C* = 0.0031Q + 0.1675 (= 0.9983) (7)

*Q/C* = −0.0119*Q* + 0.7274 (= 0.9832) (8)

Their *K*_d_ were calculated to be 322.58 µmol/L and 84.03 µmol/L, and *Q*_max_ was 54.03 μmol/g and 61.13 μmol/g, respectively. Two linear regression equations suggested that two different binding sites for sulpiride were formed in the MIP, known as specific and non-specific binding sites from imprinting cavities and residual monomer. In contrast, *Q*_max_ of the NIP was less than 19.40 μmol/g, and its non-specific adsorption capacity was also greatly decreased compared to MMA-based NIP (47.85 μmol/g) [23].

#### 3.3.2. The Kinetic Adsorption Behavior of the MIP

**Figure 5:**
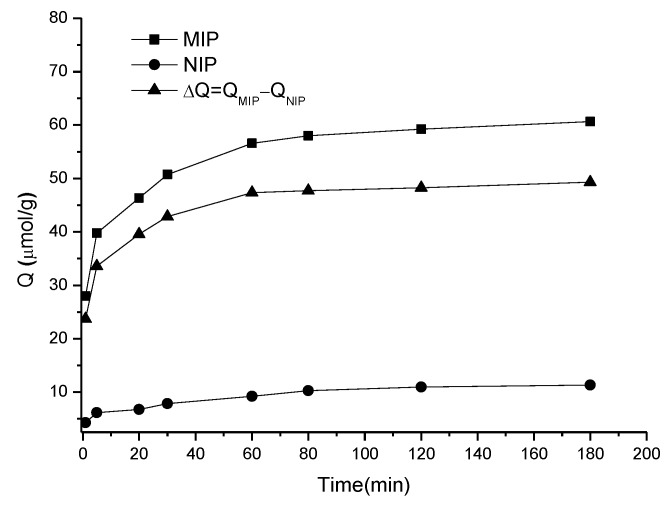
Kinetic adsorption curves of the MIP and NIP to sulpiride.

The kinetic adsorption curves of the MIP and NIP were established by monitoring the concentration of sulpiride during a period of time while the MIP and NIP were placed in sulpiride methanol solution. The results in Figure 5 show that the adsorption capacity of the MIP increased very quickly in the first 5 min, and the adsorption equilibrium was achieved at about 60 min. From 60 min to 180 min, its adsorption capacity was almost unchanged, indicating that the adsorption of sulpiride on the MIP was saturated and stabilized. In contrast, the adsorption capacity of the NIP was low and steady during the whole time. The specific adsorption Δ*Q* increased with time, indicating the effectiveness of imprinting sulpiride. The results revealed that the MIP can be used as a carrier for adsorption and extraction of sulpiride.

#### 3.3.3. The Adsorption Selectivity of the MIP 

In order to evaluate the adsorption selectivity of the MIP to sulpiride, its structural analogs, amisulpride, tiapride, lidocaine and cisapride, were chosen as substrates for the comparative study. According to the procedure of the static adsorption experiment, every substrate at 1.0 mmol/L with 2.0 mL was mixed with the MIP for its adsorption selectivity experiment. The adsorption amounts of the MIP and NIP to sulpiride and the analogs were determined by UV spectrophotometry. Then, the specific absorption ratio, imprinting factor *α* and specific factor *β* were calculated (Table 1).

In Table 1, the MIP had relatively high adsorption ability to sulpiride and its analogs compared to that of the NIP. Moreover, sulpiride had the highest selectivity among structural analogs, demonstrating more specifically imprinted on the MIP. The higher was the similarity in chemical structure, the stronger was the selective adsorption. For amisulpride and tiapride, their selectivity and capacity was attributed to the high level similarity of N-contained functional groups and molecular size as well as shape, providing evidence of amide and/or amine interaction with monomer through hydrogen bonding [39]. In addition, the steric structure of the N-contained functional group also played a role in selective binding. Compared to sulpiride, amisulpride and tiapride have one and two steric changes, respectively, affecting steric selection in binding sites. Following steric change affecting the interaction of monomer functional group with target compound, specific factor *β* might be the best parameter for evaluation of selectivity of the MIP. This comparative study exhibited the ability of specific recognition was restricted and affected by the structural matching degree. As result, imprinting factor *α* of 5.36 and specific adsorption ratio of 81.3% proved that the MIP had good specific recognition to sulpiride. Thus, it could be used for the extraction and enrichment.

### 3.4. Investigation of the Recognition Mechanism of the MIP

Substrate studied above did not provide enough evidence supporting amide and/or amine binding and also did not exclude possible binding of other functional groups which might also contribute to selective binding. Based on chemical structure, sulpiride may be conceived as a combination of three parts: p-toluenesulfonamide, formamide and 1-methylpyrrolidine. Accordingly, p-toluenesulfonamide, formamide and 1-methylpyrrolidine were used as model substrates to mimick individual functional groups for further investigation of binding sites through static adsorption experiments. Formamide and 1-methyl pyrrolidine were determined by GC and p-toluenesulphonamide was determined by HPLC. Table 2 lists selective adsorption results of the MIP to p-toluenesulfonamide, formamide and 1-methylpyrrolidine, providing correlation of functional groups in terms of selective binding. The results demonstrated that the adsorption capacity decreased in the following order: 1-methylpyrrolidine > formamide > p-toluenesulfonamide. 1-methyl pyrrolidine exhibited strong binding capacity, and even seemed to be not selective in terms of imprinting factor *α*. Hereby, it is proven that binding sites basically originated from N-contained functional group of template, resulting in the strong adsorption between template and polymers. In addition, the binding capacity of p-toluenesulfonamide in both MIP and NIP is relatively weak, and it could be only steric effect.

**Figure 6:**
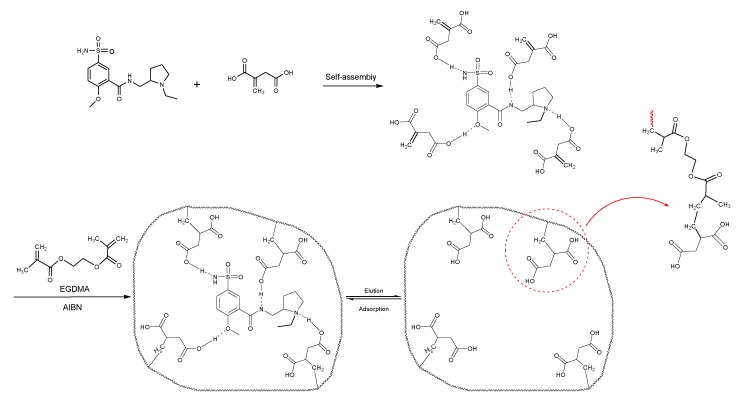
The formation process of imprinting cavity of the MIP with sulpiride.

Based on the results of sulpiride analogs and model groups, Figure 6 shows the formation of imprinting cavity of the MIP with sulpiride. It is illustrated that four monomer molecules could form interaction with a molecule of sulpiride. Only two functional groups, amide and tertiary amine, could possibly form strong binding through hydrogen bond formation, which contribute more to the selective adsorption. The other two functional groups, sulfonamide and aromatic ether, could only provide steric weak interaction due to presences of N and O. This is partially due to the rigid structure, which is lack of flexibility in the soft imprinting cavity of the MIP. There is in agreement with that 1-methyl pyrrolidine exhibits strong binding capacity but p-toluenesulfonamide did not [40,41].

### 3.5. Applications of the MIP

#### 3.5.1. Selective Solid-Phase Extraction

**Figure 7:**
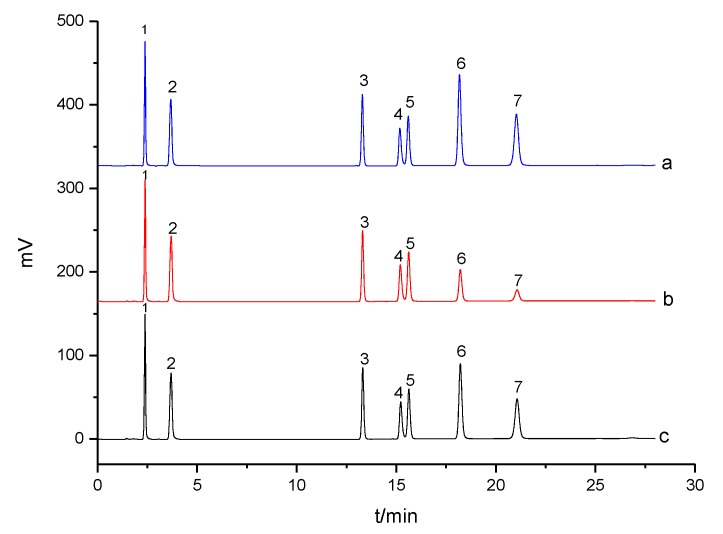
HPLC chromatograms of the mixed solution (1 μmol/L): before (**a**); and the eluent after solid-phase extraction (SPE) with the MIP (**b**); and after SPE with the NIP (**c**). 1: sulfamethoxazole; 2: sulfanilamide; 3: p-toluenesulfonamide; 4: p-nitroaniline; 5: acetanilide; 6: trimethoprim; 7: sulpiride.

To validate the selectivity of the MIP, solid-phase extraction (SPE) of sulpiride was performed by monitoring adsorption amounts of every compound in a mixed substrate solution. The mixed substrate solution consisted of different drugs, such as sulpiride, sulfonamide series (p-toluenesulfonamide, sulfamethoxazole and sulfanilamide), aniline series (p-nitroaniline and acetanilide), and heterocyclic series (trimethoprim) (as shown in Table 3) at a concentration of 0.20 mmol/L each. Figure 7 illustrates HPLC chromatograms of the mixed substrate solution before and after SPE. The special selectivity of the MIP was characterized in terms of specific factor *β*. Table 3 presents the specific adsorption of the MIP to different compounds through SPE. The results demonstrated that sulpiride was selectively imprinted in the MIP with extraction recovery of 86.9%, followed by trimethoprim, suggesting that hydrogen bonding indeed introduced strong interaction caused by monomer functional groups. Accordingly, this implied that the combination of amide and amine interaction plus additional steric effect might affect adsorption selectivity. This study also exhibited that the MIP had good specific recognition to sulpiride, and it could be used for extraction and enrichment.

#### 3.5.2. Solid-Phase Extraction of Serum Sample

**Figure 8:**
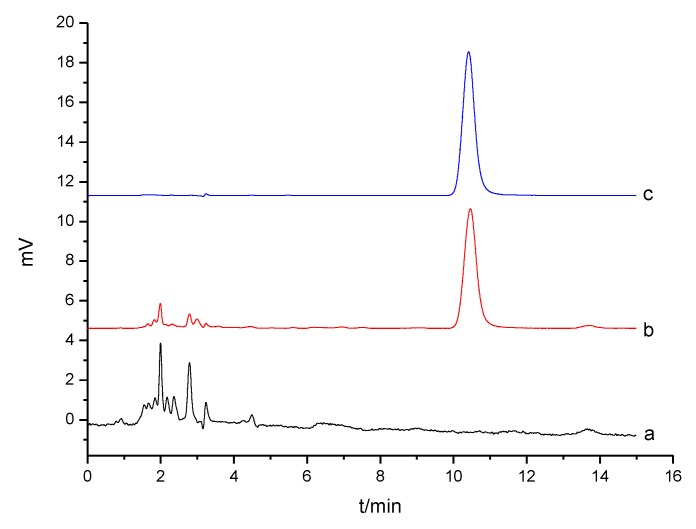
The HPLC chromatograms of: rat serum blank (**a**); rat serum sample after SPE with the MIP (**b**); and standard solution of sulpiride at 0.100 µmol/L (**c**).

Considering real application of the MIP as a solid-phase extractant [42,43], serum sample was used to investigate the effect of complex matrix on the MIP adsorption of sulpiride. Solid-phase extraction of sulpiride in rat serum was performed by the MIP adsorption (Shown in Figure 8), and recovery tests were also completed by spiking sulpiride at different levels. After elution of sulpiride adsorbed by the MIP, its concentration was determined by HPLC. As shown in Table 4, recoveries of sulpiride adsorbed by the MIP ranged from 81.57% to 86.63%. The results exhibited that there is no significant interference of serum matrix to the MIP adsorption. It is also noted that the spike level should ideally be controlled at lower than 1.00 μmol for 10 mg of the MIP, because too high a spike level could not ensure sulpiride recovery at more than 86%. As a result, the MIP could effectively extract sulpiride from the serum sample, suggesting that it can be used as a potential solid-phase extractant for preconcentration and cleanup in complex biological samples.

#### 3.5.3. Drug Release of the MIP [44]

**Figure 9:**
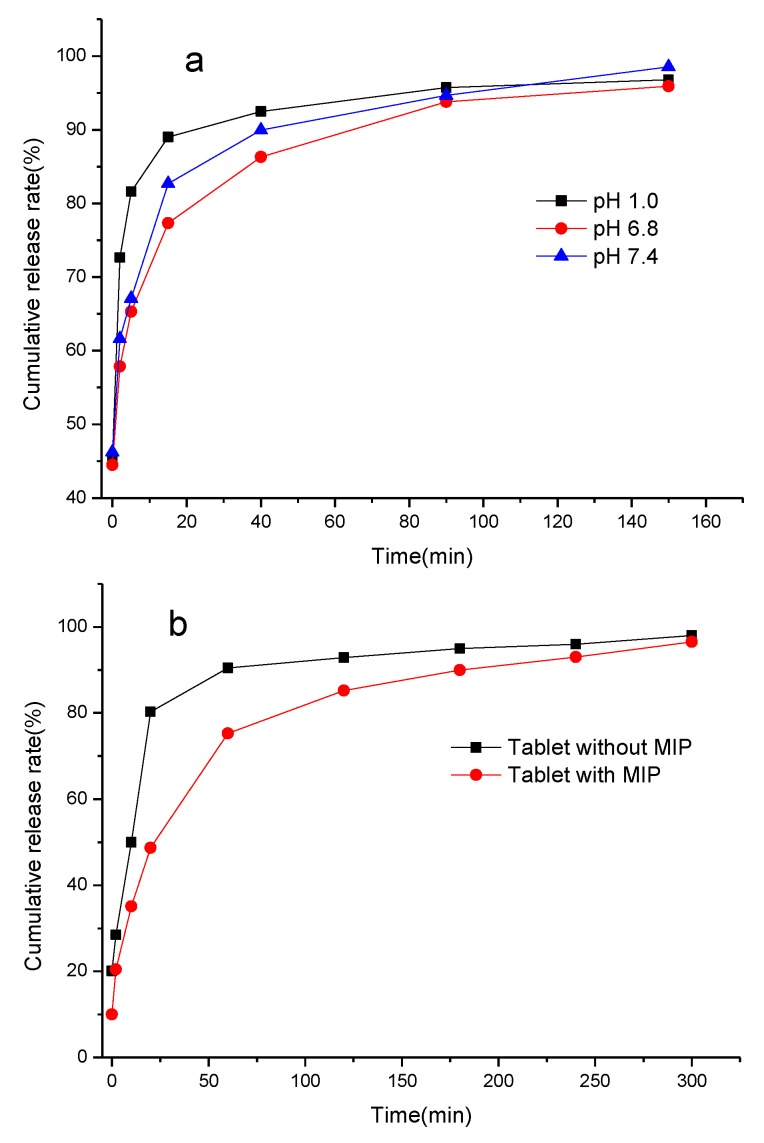
Release curves of: MIP–sulpiride in the different pH media (**a**); and sulpiride tablets with and without MIP (**b**).

The controlled release of MIP–sulpiride was carried out in simulated gastric fluid (pH 1.0), intestinal fluid (pH 6.8) and blood plasma (pH 7.4) under continuous stirring at 37 ± 0.5 °C. The results indicated that the drug cumulative release rate depends on the pH of medium. The release rate at pH 6.8 and/or 7.4 was remarkably slower than that at pH 1.0. The reason is that sulpiride as a weak base was easily dissolved in the gastric fluid, and resulted in the MIP collapsed. Clearly, the MIP (as shown in Figure 9a) at pH 6.8 and 7.4 showed a better ability of controlling drug release. This effect is ascribable to specific binding sites in the polymeric network which slowly release the drug. Thus, a MIP–sulpiride tablet was taken to conduct drug release test in simulated intestinal fluid (pH 6.8) according to the procedure described in Section 2.8. In contrast, a drug tablet consisting of identical pharmaceutical excipients was also tested. As shown in Figure 9b, the release rate of contrast tablet was up to 80.3% while only 48.7% of the drug was released from the MIP tablet. Furthermore, the tablet without the MIP was almost completely released within 60 min, whereas MIP tablet took 240 min for the release to be completed. It revealed that drug release from the contrast tablet was remarkably faster than that of the MIP tablet. The results confirmed that the MIP used as a carrier could control the release of sulpiride to a certain extent. Therefore, the MIP may be utilized as potential recognition material for controlled release, enabling sulpiride to be delivered to the targeted site in clinic applications.

## 4. Conclusions

In this work, a MIP was prepared by bulk polymerization using sulpiride as the template molecule, ITA as the functional monomer and EGDMA as the crosslinker. The MIP optimized according to template-monomer-crosslinker ratio of 1:4:15 showed good adsorption property with imprinting factor *α* of 5.36 and maximum adsorption capacity of 61.13 μmol/g. The ITA-based MIP greatly decreased the nonspecific adsorption compared to the MMA-based MIP [22,23]. The MIP demonstrated high selectivity and binding capacity towards sulpiride against the structural analogs including amisulpride, tiapride, lidocaine and cisapride. Rebinding experiments of small molecules and analogs with similar functional groups revealed the adsorption recognition mechanism of the MIP, suggesting that its high selective ability mainly depended on the binding sites from functional groups of amide and amine, which formed hydrogen bindings between sulpiride and ITA in addition to space matching. Based on the kinetic characteristic and the adsorption capacity, the MIP could provide a potential carrier material for solid-phase extraction and drug release due to the larger surface area and porosity. Accordingly, the MIP could be used to selectively extract sulpiride from a mixed solution containing different constituents (p-toluenesulfonamide, sulfamethoxazole sulfanilamide, p-nitroaniline, acetanilide and trimethoprim) prior to HPLC analysis. Moreover, the MIP, as a solid-phase extractant, was also successfully applied in serum analysis, achieving high recoveries from 81.57% to 86.63%, which meant the MIP effectively extract sulpiride from complicated serum matrix for removal of interferences. Finally, the drug release tests of a tablet loading the MIP–sulpiride exhibited that the adsorption ability of the MIP could obviously inhibit sulpiride release rate compared to a contrast. Thus, the MIP prepared was proven to be a promising alternative to solid-phase extraction and controlled release.

## Figures and Tables

**Table 1:** Selective adsorption of the MIP to sulpiride and its analogs.

Substrate	Chemical Structure	*Q*_MIP_ (μmol/g)	*Q*_NIP_ (μmol/g)	*ΔQ* (μmol/g)	Specific Adsorption Ratio (%)	Imprinting Factor, *α*	Specific Factor, *β*
Sulpiride	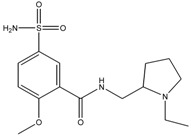	60.05	11.20	48.85	81.35	5.36	1.00
Amisulpride	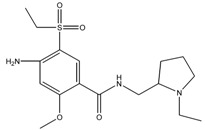	55.00	15.10	39.90	72.55	3.64	0.82
Tiapride	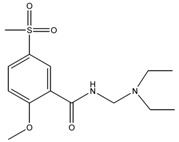	50.35	15.55	34.80	69.12	3.24	0.71
Lidocaine	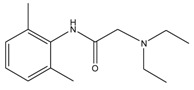	25.75	6.60	19.15	74.37	3.90	0.39
Cisapride	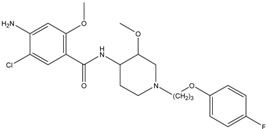	18.50	6.55	11.95	64.59	2.82	0.24

**Table 2:** The results of selective adsorption of the MIP to p-toluenesulfonamide, formamide and 1-methylpyrrolidine

Substrate	Chemical Structure	*Q*_MIP_ (μmol/g)	*Q*_NIP_ (μmol/g)	*ΔQ* (μmol/g)	Specific Adsorption Ratio (%)	Imprinting Factor, *α*
p-Toluenesulfonamide	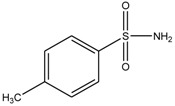	0.51	0.01	0.50	98.04	–
Formamide	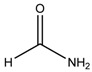	7.53	2.51	5.02	66.67	3.01
1-Methylpyrrolidine	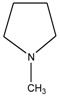	85.01	62.46	22.55	26.52	1.36

**Table 3:** The results of solid-phase extraction of different compounds with the MIP.

Substrate	Chemical Structure	*Q*_MIP_ (μmol/g)	*Q*_NIP_ (μmol/g)	Recovery (%)	Specific Factor, *β*
Sulfamethoxazole	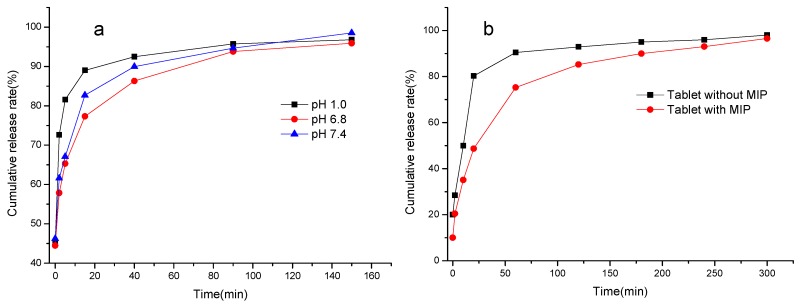	0	0	0	0
Sulfanilamide	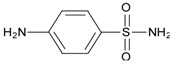	0.21	0.03	2.10	0.03
p-Toluenesulfonamide	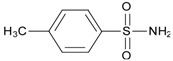	0	0	0	0
p-Nitroaniline	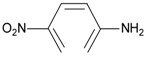	0.24	0.13	2.39	0.02
Acetanilide	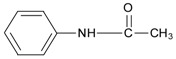	0	0	0	0
Trimethoprim	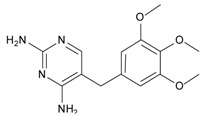	6.43	3.26	64.3	0.52
Sulpiride	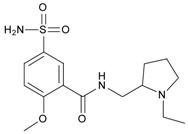	8.39	2.28	86.9	1.00

**Table 4:** The results of solid-phase extraction of sulpiride from rat serum samples by the MIP (n = 3).

Sample	Spike Levels (μmol/L)	Determined (μmol/L)	Recovery (%)
1	0.050	0.432 ± 0.005	86.63 ± 0.75
2	0.100	0.862 ± 0.038	86.23 ± 3.75
3	0.150	1.223 ± 0.041	81.57 ± 2.73
